# Cervical cancer knowledge and barriers and facilitators to screening among women in two rural communities in Guatemala: a qualitative study

**DOI:** 10.1186/s12905-022-01778-y

**Published:** 2022-05-28

**Authors:** Kristin G. Bevilacqua, Anna Gottschlich, Audrey R. Murchland, Christian S. Alvarez, Alvaro Rivera-Andrade, Rafael Meza

**Affiliations:** 1 Department of Population, Family and Reproductive Health, Johns Hopkins Bloomberg School of Public Health, 615 N Wolfe St, Baltimore, MD 21205 USA; 2 BC Women’s Hospital and Health Service, Women’s Health Research Institute, 4500 Oak St, Vancouver, BC V6H N9 Canada; 3 Faculty of Medicine, University of British Columbia, 31702194 Health Sciences Mall, Vancouver, BC V6T 1Z3 Canada; 4 Department of Epidemiology, Harvard T.H. Chan School of Public Health, 677 Huntington Ave, Boston, MA 02115 USA; 5 Instituto de Nutrición de Centro América y Panamá,, Calzada Roosevelt 6-25 Zona 11, Guatemala City, Guatemala; 6 School of Public Health, Department of Epidemiology, University of Michigan, 1415 Washington Heights, Ann Arbor, MI 48109 USA

**Keywords:** Cervical cancer, Cervical cancer screening, Barriers and facilitators, Health disparities

## Abstract

**Background:**

Approximately 80% of deaths due to cervical cancer occur in low- and middle-income countries. In Guatemala, limited access to effective screening and treatment has resulted in alarmingly high cervical cancer incidence and mortality rates. Despite access to free-of-cost screening, women continue to face significant barriers in obtaining screening for cervical cancer.

**Methods:**

In-depth interviews (N = 21) were conducted among women in two rural communities in Guatemala. Interviews followed a semi-structured guide to explore knowledge related to cervical cancer and barriers and facilitators to cervical cancer screening.

**Results:**

Cervical cancer knowledge was variable across sites and across women. Women reported barriers to screening including ancillary costs, control by male partners, poor provider communication and systems-level resource constraints. Facilitators to screening included a desire to know one’s own health status, conversations with other women, including community health workers, and extra-governmental health campaigns.

**Conclusions:**

Findings speak to the many challenges women face in obtaining screening for cervical cancer in their communities as well as existing facilitators. Future interventions must focus on improving cervical cancer-related knowledge as well as mitigating barriers and leveraging facilitators to promote screening.

## Background

Cervical cancer is a highly preventable disease. Despite this, there were an estimated 604,127 new cases and 341,831 deaths due to cervical cancer globally in 2020, making it the fourth most common cancer among women [1]. Cervical cancer incidence and mortality disproportionately burden women in low- and middle-income countries (LMICs), reflecting a lack of access to effective screening, vaccination and treatments that enable both preventive and curative care. In 2020, the incidence rate of cervical cancer in LMICs was 18.8 per 100,000 women versus 11.3 in high income countries (HICs) and the mortality rate in LMICs was more than twice that of HICs (12.4 per 100,000 women vs. 5.2 per 100,000 women) [1].

Guatemala is one such nation where limited access to effective screening and treatment has resulted in an alarmingly high cervical cancer incidence rate of 20.3 per 100,000 and a mortality rate of 11.9 per 100,000 women in 2018 [2]. Surprisingly, given the high burden of cervical cancer in the country, cervical cancer screenings are provided free-of-cost through the Guatemala Ministry of Health (MOH) [3], both through local women’s health clinics and contracts with non-government organizations (NGOs) [4]. In the early 2000s, NGOs in Guatemala introduced visual inspection with acetic acid (VIA) and cryotherapy as an alternative to cytology-based screening (i.e. Pap smears) [4]. VIA was formally integrated into the national cervical cancer screening program in 2008 and now accounts for the majority of cervical cancer screenings in the country [4]. In 2015, the MOH launched a pilot implementation trial for human papillomavirus (HPV) testing in urban settings to evaluate its feasibility as an alternative to traditional screening methods [5].

Despite these services, approximately 64% of women in Guatemala report lifetime cervical cancer screening, with lower rates among indigenous and rural women [6]. This disconnect suggests the need for a more nuanced exploration of the barriers women in Guatemala face in obtaining cervical cancer screening. Extant literature in other LMIC settings suggests that limited cervical cancer knowledge [7], fear of the procedure [7] and possible positive test results [8], embarrassment [7, 9] and competing costs [8, 9] present significant barriers to cervical cancer screening. In Guatemala in particular, cost and distance, permission required to attend, not wanting to attend screening alone, and language discordance with providers were found to be associated with never being screened [6]. In addition to these barriers, even if women are able to access screening, its effectiveness in reducing cervical cancer incidence and mortality further depends upon access to follow-up care and treatment, which are also often lacking [10].

In August 2020, the World Health Assembly adopted the Global Strategy for the elimination of cervical cancer to reduce the age-adjusted incidence rate of cervical cancer to less than 4 per 100,000 women-year in all countries by the end of the century through the promotion of HPV vaccines, screening, and treatment [11]. In light of this global effort, a better understanding of the reasons why free-of-cost screening in Guatemala has not resulted in improved cervical cancer outcomes is warranted. To our knowledge, the present study is the first to utilize in-depth, qualitative interviews to elucidate barriers and facilitators to cervical cancer screening among women in two rural communities in Guatemala.

## Methods

### Study setting

#### Santiago Atitlán, Sololá

Santiago Atitlán (Santiago) is a majority Maya-Tz’utujil community on the southern shore of Lake Atitlán. Santiago’s roughly 45,000 inhabitants live in 20 *cantones* or neighborhoods, located in the town’s center and spreading out to more rural, surrounding neighborhoods. Nearly all of Santiago’s inhabitants speak Tz’utijil as their first language and Spanish as a second language. A small proportion speak only Tz’utujil [12].

#### Livingston, Izabal

Livingstonl is situated on the Caribbean coast of Guatemala and is accessible only by boat. Livingston is an ethnically diverse community with a large Garifuna (Afro-Caribbean) population as well as Maya-Q’eqchi and *Ladino* (mixed-race) populations. Most inhabitants speak Spanish and either Garifuna or Q’eqchi [13].

### Study population

Participants were recruited from a larger pool of women who had been sampled to participate in the HPV multi-ethnic study (HPV MES), which involved completing a quantitative survey on prior screening behavior and acceptability of Human Papillomavirus (HPV) self-collection sampling to screen for cervical cancer, as well as the opportunity to participate in self-collection HPV-based screening [13]. In brief, participants in Santiago were randomly sampled through stratified multilevel sampling based on maps and population counts. Due to lack of census data, participants in the larger study in Livingston were recruited through convenience sampling. Details and main results of this study are described elsewhere [13, 14]. Following completion of the quantitative survey, women consented to be contacted for an in-depth, semi-structured interview. Interview participants in both communities were sampled purposively by neighborhood.

### Community partners

The study team worked collaboratively with community partners at both study sites. In Santiago, the team worked with local health clinic, Rxiin T’Namet, which provides family and reproductive health services to residents, including workshops and community outreach. In Livingston, research was supported by a local HIV-focused health clinic, Iseri Ibagari. The clinic provides HIV testing, referrals for treatment, and health education workshops focused on reproductive health. Both community partners helped to publicize the study to the larger community and provided private spaces for in-depth, semi-structured interviews.

All study procedures were approved by the Institutional Review Boards at the University of Michigan (HUM00096559) and the Instituto de Nutrición de Centro America y Panamá (INCAP) (MI-CIE-16-009), located in Guatemala City.

### Data collection

Interviews were conducted privately in the women’s homes or spaces provided by community partners. Interviews were conducted in Spanish by the lead qualitative investigator and began with the administration of informed consent in the participant’s preferred language. An interpreter provided real-time translation for participants in Santiago Atitlán who completed the interview in Tzu’tujil. Interviews lasted 60–90 min and followed a semi-structured interview guide to better understand the barriers and facilitators to cervical cancer screening faced by participants (Table 1). Interviews were conducted until common themes suggested data saturation as it related to our major research questions. All interview were recorded and transcribed verbatim by native Spanish speakers. Those conducted in Tzu’tujil were translated and transcribed into Spanish by a native Tzu’tijil speaker.

**Table 1:** Sample items from the semi-structured interview guide

Can you tell me a little bit about cervical cancer?	
Where did you get this information from?	
Before today, had you ever spoken with a doctor about cervical cancer screening?	
Can you tell me a little more about that conversation?	
Have you ever been screened for cervical cancer?	
In general, how was your experience?	

### Analysis

Utilizing an adapted Framework Analysis approach [15], the transcribed data were analyzed by three Spanish-speaking study team members who independently reviewed five transcripts for emergent themes that related to the original research questions. Codes were created to characterize emergent themes and revised in an iterative process to ensure coverage and reliability between coders. The resulting final codebook included 56 codes, their definitions, and examples of each code (“Appendix A”). The remaining 16 transcripts were each coded by two of the three Spanish-speaking study team members; coding inconsistencies were reconciled by the lead qualitative investigator and organized using NVivo 9 (QSI International). Data were then organized by theme using an analytic matrix, which was reviewed by the lead investigator and a fourth Spanish-speaking study team member. Illustrative quotes were selected relating to salient themes and subthemes and translated into English. Translations were reviewed by two native-Spanish speaking members of the research team for consistency with their original significance.

## Results

Twenty-one women (Santiago Atitlán, n = 10; Livingston, n = 11) were interviewed. Participant demographics, stratified by community, are provided in Table 2. Women in Livingston had higher average household incomes, educational attainment, and literacy. Marriage rates in both communities were comparable. About 80% of women in both communities reported having ever been screened for cervical cancer.

**Table 2:** Demographic characteristics by study component and study site

	Survey participants			Interview participants		
	Total (N = 956) n (%)	Santiago (N = 500) n (%)	Livingston (N = 456) n (%)	Total (N = 21) n (%)	Santiago (N = 10) n (%)	Livingston (N = 11) n (%)
Age, mean (SD)	33.92 (9.45)	34.78 (8.44)	32.97 (10.38)	35.33 (9.17)	36.50 (8.95)	34.27 (9.68)
*Ethnicity, n (%)*						
Tz’tujil	483 (50.42)	483 (96.60)	0 (0)	10 (47.62)	10 (100)	0 (0)
Ladino	122 (12.76)	9 (1.80)	113 (24.78)	4 (19.05)	0 (0)	4 (36.36)
Garifuna	145 (15.27)	0 (0%)	145 (31.80)	6 (28.57)	0 (0%)	6 (54.55)
Q’eqchi’	191 (19.98)	0 (0%)	191 (41.89)	1 (5.76)	0 (0%)	1 (9.10)
Other	15 (1.57)	8 (1.60)	7 (1.54)	0 (0)	0 (0%)	0 (0)
Literate, n (%)	653 (68.31)	255 (51.00)	398 (87.28)	15 (71.43)	5 (50%)	10 (90.91)
*Education, n (%)*						
Less than primary	500 (52.30)	347 (69.40)	153 (33.55)	8 (38.09)	2 (18.18)	6 (60)
Primary/secondary	255 (26.67)	100 (20.00)	155 (33.99)	7 (33.33)	5 (45.45)	2 (20)
More than secondary	193 (20.19)	50 (10.00)	143 (31.36)	6 (28.57)	4 (36.36)	2 (20)
Unknown	8 (0.84)	3 (0.60)	3 (1.10)	0 (0)	0 (0)	0 (0)
Married/United	683 (71.44)	431 (86.20)	252 (55.26)	12 (57.14)	6 (60)	6 (54.55)
Ever screened (Pap/VIA), n (%)	602 (62.97)	337 (67.40)	265 (58.11)	17 (80.95)	8 (80)	9 (81.81)
Screened in past year, n (%)	235 (24.58)	101 (20.20)	134 (29.39)	8 (38.10)	2 (20)	6 (54.55)

### Cervical cancer and screening knowledge and beliefs

Cervical cancer knowledge discussed in qualitative interviews was variable across sites and across women. Overall, women in Livingston shared greater cervical cancer knowledge than women in Santiago, though several women at both sites reported no cervical cancer-related knowledge. This knowledge difference is supported by previously published quantitative findings among the larger HPV MES sample, which found higher knowledge of HPV and perceived severity of cervical cancer in Livingston [13]. In Livingston, the majority of interview participants had heard of cervical cancer and several women reported knowledge related to cervical cancer development and symptomology, including that it is often asymptomatic, resulting in later-stage diagnoses and poor prognoses for women who are not regularly screened.

*I had heard of cancer of the neck of the womb [cervix], that if someone is not periodically getting their Pap smear…you can’t detect it because it’s silent…it has no symptoms and then it’s there, so you didn’t realize, and it’s already advanced significantly. (Livingston, Garifuna, Age 46–50, Screened)*

In comparison, no knowledge of cervical cancer as well as misinformation related to cervical cancer was more commonly reported in Santiago. For example, several women shared beliefs about the causes of cervical cancer that were not related to sex or to HPV, including that it is caused by sadness or worry, bad food, or lack of vitamins. While sharing her desire to learn more about cervical cancer one women asked:

*[…] I want to know more about where this illness comes from. Let’s say my daughter, some days she’s fine but other days her stomach hurts. That’s why I ask, how does this illness start […] could it be that you get sick a lot…or you have a certain diet? I don’t know. That’s just what I have heard. (Santiago, Tzutujil, Age 46–50, Screened)*

Poor hygiene or a lack of personal care was also commonly cited as a cause of cervical cancer.

*There are cases in which they say that all of a sudden the cancer appears in the neck of the womb [cervix] when you don’t take care of yourself or don’t have good hygiene. (Santiago, Tzutujil, Age 40–45, Screened)*

Women at both sites, including those self-reporting lower levels of knowledge spoke of the connection between cervical cancer and sex, though few mentioned HPV. Many discussed this relationship in terms of male partners engaging in extra-relational sex with other women or with sex workers, as well as women themselves having multiple sex partners. In turn, women discussed the need to “cuidarse” or take care of oneself within the context of sexual relationships. When asked what it mean to take care of oneself in relation to cervical cancer, one Santiago participant explained through an interpreter:

*She has heard that to take care of herself, she shouldn’t sleep with other men. Because she heard that when men have sexual relations with a lot of women…it’s possible that you get infected with this illness. (Santiago, Tzutujil, Age 30–35, Never-screened)*

Knowledge related to cervical cancer screening also differed greatly across participants. In both Santiago Atitlán and Livingston, several women reported no knowledge of cervical cancer screening but familiarity with Pap smears specifically, suggesting that women may obtain screening without understanding its purpose. When asked if she had heard of a test to detect cervical cancer, one participant in Santiago responded:

*I don’t know of one. I don’t know simply because we subject ourselves to any of that. (Santiago, Tzutujil, Age 30–35, Never-screened)*

However, when asked if she had heard specifically of Pap smears, she responded:

*Yeah, I have heard, well they told me that it’s a small apparatus that is introduced to a women’s internal part where they examine her.*

Women posited that lack of knowledge related to cervical cancer and screening in their communities was tied to limited sexual education informed by cultural taboos around sex and reproductive health.

*There are teachers that see this as taboo and so they don’t want to speak openly about sexuality, few speak about it or see it as something normal, like a normal part of life […] so maybe of the 100%, 45% have the freedom to speak this way, openly, about sexuality, but others, they are limited. (Livingston, Garifuna, Age 26–30, Screened)*

Despite these taboos, informal and formal conversations (e.g., community trainings or *charlas*) between women were an important source of knowledge. Speaking about her friend who encouraged her get screened, one Livingston women explained:

*Because she is also open, because there are people who are embarrassed to talk about these things, like they are very private sometimes, but with her, I don’t have any problem talking with her. (Livingston, Garifuna, Age 46–50, Screened)*

### Barriers to cervical cancer screening

Women across both communities expressed similar barriers to obtaining cervical cancer screening. Barriers generally fell into one of three, often intersecting, categories: individual, interpersonal and system-level. At the individual level, cost was the most commonly cited barrier. Though all women in Guatemala are entitled to free cervical cancer screening through publicly run women’s health clinics, in practice ancillary costs including transportation, food, and loss of income pose significant barriers to screening.

*There’s a shortage of money…You go to a [women’s clinic] and you pay for a taxi, you pay for a pick-up truck and sometimes people just can’t do it and then, because of this they say, “better not, I have to save up to go there, better not, and if I go and they don’t see me, better to not go”. (Santiago, Tzutujil, Age 26–30, Screened)*

Women also perceived the cost of future tests and treatment that may result from a diagnosis as a barrier to seeking screening. When asked why she had never been screened, one participant in Livingston explained:

*Maybe because it requires money and also if one suffers from an illness that’s what happens, because people have many illnesses and sometimes they tell you, you have to buy this medicine now and you do not have money, I cannot buy it. (Santiago, Tzutujil, Age 36–40, Screened)*

Women understood this both abstractly and from personal experience. Among those who had previously received screening, two women shared their own stories of being unable to afford treatment after a positive screening. One participant from Livingston recalled being unable to afford a biopsy after doctors found a lesion on her cervix during a routine Pap smear:

*Well, in fact I got a video colposcopy because according to the results, I have […] an ulcer on my cervix. I got a video colposcopy […] and the doctor explained everything to me, that I have to do another test to make sure it doesn’t become cancerous, you have to detect it early, so it doesn’t become cancer. But with my economic situation…for me I can’t afford more tests. (Livingston, Ladino, Age 26–30, Screened)*

At the interpersonal level, several women cited male partners as a potential barrier to obtaining screening among women, citing a culture of “machismo” in which women are seen as subordinate to men [16]. This barrier was twofold: men who exert economic control over their female partners may refuse to provide the money for screening or male partners may withhold permission, explicitly or tacitly, for their female partners to obtain a screening.

*Because there are men who say no, because of machismo…and the women also say, “my partner doesn’t want me to,” so no. Women are scared that they [the men] will hit them or leave them. (Santiago, Tzutujil, Age 30–35, Screened)*

For other women, this control was more subtle. After experiencing extreme abdominal pain, one women’s mother insisted she obtain a Pap smear:

*I didn’t want to go, but my mom came and told me, “let’s go because this…is going to kill you”. So I went with [my family] to get the exam, but it was already late and my husband was about to come back from the mountain and I got very worried because we didn’t have money, and he didn’t know anything and no one told him anything. I went that one time in a hurry and that was the only time I have done [a Pap smear]. (Santiago, Tzutujil, Age 40–45, Screened)*

Women described how knowledge acquisition related to cervical cancer was also informed by gender norms.

*I also think it’s machismo. The majority of women live with machista men, they demand order, food, everything in the house. So I think this also influences the lack of knowledge. (Livingston, Garifuna, Age 26–30, Screened)*

Fear was also a barrier commonly expressed by women in both communities. At the individual level, fear of pain or discomfort during the screening itself was common among screening-naïve women.

*[I have not been screened] well, out of fear, why lie to you. I have this fear, I can’t explain it exactly…but every time that I have to do the Pap smear, they notify me, but I always end up refusing. The fear about the Pap smear, how do I explain it, what I am scared of is the instrument. (Livingston, Garifuna, Age 26–30, Never-screened)*

*[…] they say that it hurts, that they insert, I don’t know what, a tube that, yes but when I went when I saw, I realized that no…that it wasn’t like that. (Livingston, Garifuna, Age 26–30, Screened)*

Women in both communities expressed fear of embarrassment or *vergüenza* of being seen by a doctor or other healthcare worker. Notably, this fear often presented a barrier to seeking screening even when free-of-cost screening was available. Often this fear centered on the need for a healthcare provider to view intimate body parts to conduct the screening.

*I’m embarrassed. I don’t know why, my daughters have told me, “Listen this is where they are providing Pap smears for free, why don’t you go mama. You are sick. It’s deadly. You have said yourself you have never taken care of these things…maybe you have a bad stomach”. But I say I have decided to die this way…I don’t want [doctors] to see me, I say. […] So it happens that I have never done [a Pap smear]. (Livingston, Ladina, Age 50–55, Screened)*

Women in Santiago expressed similar concerns:

*Before, in Santiago Atitlán, women almost never saw the doctor because of embarrassment of being seen [nude]. Because the women only want to be seen by their husbands and no one else. Because it would be an ugly thing for them to be seen by someone else so they, more than anything, feared [the embarrassment]. (Santiago, Tzutujil, Age 26–30, Screened)*

Fear of disclosure of personal information was another barrier. In both communities, women cited personal experience or experiences among their friends and family in which their medical confidentiality was violated. This fear stemmed, in part, from the fact that healthcare workers were often well known in the community or even related to their patients.

*Well, uh, when people know each other, it’s uncomfortable because, imagine that they’re looking at you, there are people who do not have professional ethics and they are talking about your vagina and all of these things, but when there are people that don’t know you, that’s not an issue, there is security. (Livingston, Garifuna, Age 26–30, Screened)*

To avoid potential violations of their privacy, women at both sites traveled to other towns to obtain screenings.

*Well, because I think that at times here at the health center you lose a little bit of ethics, then because we all know each other, already there isn’t much confidence in this sense…if one knows the things going on with a patient, it doesn’t need to come out…it needs to be something confidential…because this has to do with someone’s willingness to do [a screening]. Because if one sees that there is no formality, then what for. Better I don’t do it or I find somewhere else to do it. (Livingston, Ladina, Age 20–25, Screened)*

Further, general concerns about treatment by providers presented a barrier to screening for many women. In Santiago in particular, indigenous-identifying participants described experiences of discrimination when obtaining screening at the local health center.

*[…] after a while laying down my vagina started to hurt. I had a Pap smear not long before and it did not hurt much but that day it hurt a lot. The speculum wouldn’t go in, it doesn’t reach the uterus maybe…They thought that I didn’t understand Spanish, so she was explaining to the other nurse, “the speculum won’t go in what are we going to do”... (Santiago, Tzutujil, 30–35, Screened)*

When asked how this experience made her feel she responded:

*[It made me feel] bad, I asked the nurse why she didn’t put the speculum in. “No it went it. It reached the uterus,” she told me. But I heard what they were saying because I was laying there with my legs open. But I was angry with myself. If I had done it in another place, it would have been better to pay…it’s free, but as they say, what’s free comes at a cost.*

At the system level, resource constraints were commonly cited by women in both Santiago and Livingston. Despite access to government subsidized screenings at public health clinics, women cited shortages of tests and long wait times.

*Suppose there at the [public women’s health clinic] one goes early, at six in the morning…and you leave at eleven or twelve in the afternoon. It’s a long time for someone, and you have to be cooking and doing things in the house. (Santiago, Tzutujil, 30–35, Never-screened)*

Due to shortages, women often sought screenings through traveling health campaigns or "jornadas"; however, these options also presented challenges. When describing the process for obtaining a free screening at health campaign in Livingston, one participant explained:

*You could say, the woman who gets there first, gets the test. Because when they come, when they give screenings, they only bring tools for a certain number of people…if they only bring enough for 50 people, only 50 people will be seen…you have to wait until the next time they come back…it’s stressful because...sometimes you don’t sleep because you’re thinking about waking up the next day early to go running [to get screened]. (Livingston, Garifuna, Age 25–30, Screened)*

Barriers to obtaining a free or low-cost screening in turn, create a tiered system in which those women who could afford to pay out of pocket for a screening at a private clinic, were more likely to seek care. Speaking about healthcare more broadly, one woman in Santiago Atitlán explained:

*I don’t go because I don’t have much money. In the [private hospital], it’s not the same as going to the [public women’s health clinic]. At the [public women’s health clinic] they don’t charge, but there aren’t specialists. There are times when for this reason people don’t go to the doctor to see what illness they have, until they are even more sick. (Santiago, Tzutujil, Age 25–30, Screened)*

### Facilitators to cervical cancer screening

Despite significant barriers to screening, a large proportion of our sample reported being screened at least once in her lifetime and women described several facilitators to screening. In both communities, the desire to know one’s health status motivated women to obtain screening.

*Well, I thought, if I don’t do it, I’ll get worse. I have heard that there are many young women who do [the screening] quickly to stay clean, and that’s why I thought to do [the screening] before something bad happens to me (Santiago, Tzutujil, Age 45–50, Screened)*

Among some women who had obtained previous Pap smears, screening was often precipitated by symptoms or other health concerns.

*What happened was I had a miscarriage, or they had to do a curettage because all of a sudden my bleeding was really, really heavy so they sent me to do a curettage but they told me first they were going to do a Pap smear. (Livingston, Ladina, Age 25–30, Screened)*

*The first time I did [the Pap smear] was because my stomach hurt really bad, so I said, “I don’t know what I have” because it hurt a lot and I had a lot of discharge. It hurt constantly so I went, that’s why I went to see what I had. (Santiago, Tzutijul, 25–30, Screened)*

Women also described interpersonal and system-level facilitators that promoted cervical cancer screening. Though not consistent across participants, some women expressed that provider-communicated screening guidelines prompted them to obtain screenings. When asked how she knew to obtain her first screening, one Livingston resident replied:

*Well, in general how I told you, through information, through the [local health clinic] because sometimes one goes there…they explained to me what it was about, why it was good to do it and because of that, I had no problem [getting screened]. (Livingston, Garifuna, 45–50, Screened)*

At the system level, health campaigns, or “jornadas”, outside of the public health system also facilitated screening. Women often traveled to nearby towns hosting health campaigns for a variety of health services. However, as discussed above, several women described health campaigns as irregular, lacking follow-up and often tied to some other event. In one extreme example, a participant from Santiago obtained her first and only Pap smear during the aftermath of Hurricane Stan in 2005, which resulted in mudslides that killed over 1000 residents.

*I got my Pap smear after the accident caused by Hurricane Stan. That day, they set up shelters for people and they came from the health center to do a health campaign and I took advantage to get my Pap smear. (Santiago, Tzutujil, 30–35, Screened)*

In this way, such health campaigns may facilitate access for individual women, but their irregularity may also present barriers to care if they are women’s only screening option.

## Discussion

Cervical cancer is a major public health concern for women in LMICs, where barriers to screening often prevent early diagnosis and treatment [6, 17, 18]. The present study sought to examine the cervical cancer knowledge and barriers and facilitators to screening among a sample of women in two rural communities in Guatemala. Barriers and facilitators at the individual, interpersonal, and system levels were identified by women in both communities.

Limited knowledge related to cervical cancer was common but differed across women and site. These findings are in line with previous quantitative and qualitative research in Guatemala [19] and other LMIC contexts [20–22]. Notably, while some women reported no knowledge of cervical cancer screening in general, they were aware of and, in some instances, had obtained screening. This discordance suggests that women may be screened by healthcare providers during other health care visits without adequate explanation of the purpose of the procedure or its risks. This is congruent with other provider-related interpersonal barriers to screening reported by women in our sample, including poor communication, experiences of discrimination and distrust of healthcare providers as well as earlier research which found patient/provider language discordance to be a barrier to screening [6]. In contrast, community health workers (CHWs) and workshops were identified by women as facilitators to screening. CHWs have been found to be effective in increasing cervical cancer knowledge among women in other LMIC settings [23] and task shifting from physicians to CHWs has been found to be an effective strategy for the management of non-communicable diseases, including screening [24]. Our results suggest that interventions to improve screening may include promoting the integration of CHWs in clinical settings to facilitate patient-provider communication and to ensure women understand screening recommendations and the risks and benefits of cervical cancer screening.

Resource limitations were also a major barrier to screening for most women. Despite the availability of free-of-cost screening to women in Guatemala through government-funded women’s health clinics, few women reported being able to access these services due to the associated costs of travel or food, barriers that have been identified in other LMIC settings [8, 9, 25, 26]. Cost of future procedures associated with a positive screening was also a concern and several women described personal experiences in which they were unable to afford follow-up care associated with a positive screening. Gender norms related to economic control also presented a barrier to women whose male partners were unwilling to provide needed funds to obtain screening. Interventions to limit ancillary and future costs, including minimizing the need for and/or subsidizing the cost of travel, must be considered when developing future screening programs to promote utilization of free or low-cost screening and to reduce help minimize potential interference by partners.

At the system level, shortages of materials, long wait times and irregularities in service also hindered women’s ability to access government subsidized services. Through formal agreements with the Guatemalan government, non-governmental organizations (NGOs) provide a significant proportion of healthcare services throughout Guatemala, including approximately 15% of cervical cancer screenings [4]. Though health campaigns implemented by NGOs helped supplement governmental programs, irregular scheduling, shortages of tests and similar cost-related barriers also plagued these programs. As such, the implementation of less resource-intensive screening methods [5] by governmental programs and NGOs is an important step to addressing these barriers.

Despite significant barriers to screening, most participants reported at least one previous cervical cancer screen and women identified several important facilitators to screening, including internal motivation to maintain one’s health, health campaigns and formal or informal conversations with other women. Programs to improve cervical cancer screening should leverage women’s own desire for good health and the power of interpersonal relationships in fostering health promotive behaviors, while reducing financial and logistical barriers to screening. For example, at-home self-screening for HPV has been proposed as one method to improve cervical cancer outcomes in LMICs, as this type of screening can assuage barriers such as cost (test can be performed in home) and embarrassment (test can be performed by in private, by women themselves) [14].

## Limitations

The findings of this study should be understood in the context of its limitations. Women who chose not to enroll in the larger HPV MES cohort and those who did enroll but chose not to participant in the qualitative interview may be systematically different from those who agreed to participate. With 21 interviews, our sample is relatively small; however, interviews were conducted until saturation was reached [27] and guidelines suggest that 20 interviews may be sufficient for qualitative convenience samples [28]. Social desirability bias may have impacted women’s responses during interviews. The sensitive nature of the interview was described to participants during recruitment and steps taken to ensure participant privacy were explained in detail at the start of each interview and throughout conversations.

## Conclusions

The World Health Organization has identified the “robust understanding of the social, cultural, societal and structural barriers to the uptake of services” as a strategic action necessary to achieve the elimination of cervical cancer [29]. This study is the first to our knowledge to qualitatively examine cervical cancer knowledge and barriers and facilitators to screening among women in two rural communities in Guatemala. Our findings speak to the many challenges women face in obtaining screening for cervical cancer in their communities but also to existing facilitators that may be leveraged for future interventions. Future research must focus on mitigating barriers through education and alternative screening methods.

## Data Availability

The datasets used and/or analyzed during the current study available from the corresponding author on reasonable request.

## Appendix A

**Table:**

Code	Subcode	Code abbreviation	Definition of code	Example
*Screening experience*		SE		
	No experience	SE_NO	Any mention of participant having no past experience being screened for cervical cancer	K: Y ¿como sabe uno que va a venir una jornada? E: porque empiezan a pegar carteles, y en esos mismos carteles se dicen que lugar, hora y fecha se van a hacer el Papanicolaou K: Y ¿se ha realizado usted un Papanicolaou? E: No, no me lo he realizado. Esta vez en esta jornada quiero realizarme K: ¿con el vale? E: con el vale
	Past experience	SE_PAST	Any mention of participant having past experience being screened for cervical cancer	E: Bueno yo fui a realizármelo en el hospital que se llama APROFAM. Que se especializa bastante en lo que es la mujer y todo… Entonces ahí me lo fui a realizar, me atendió una enfermera, y me atendió de lo más bien. Me explicó que era lo que me iban a hacer y todo. que es lo que yo iba a sentir. Entonces para mí, la primera vez que me lo realicé… estuvo bastante bien a lo que yo me imaginaba
	Unsure of experience	SE_UE	Any mention of participant being unsure of having been screened for cervical cancer in the past	K: ¿Ha escuchado de unas pruebas de detección para este cáncer… del cuello de matriz? E: No K: ¿Ha escuchado del Papanicolaou? E: Sí, del Papanicolaou si
	Positive experience	SE_POS	Any mention of participant having a positive experience while being screened for cervical cancer	Entrevistada: Venía más abajo. Ajá. Y entonces el doctor me dijo, puede ser que tienes un problema allí en la matriz. Mejor te voy a sacar, te voy a hacer un papanicolaou si no te molesta o estas de acuerdo, me dijo. Entonces yo le dije, ¿qué es eso?, nunca me han hecho eso. “Es solo que te vamos, te va a doler un poco, pero es normal. A toda mujer le hacen eso” así me dijo el doctor. Ah bueno. Entonces sí es para que mejore mi salud entonces sí estuve de acuerdo con el doctor. Entonces el doctor me hizo el papanicolaou pero, eh, no me dolió nada. No sentí nada
	Negative experience	SE_NEG	Any mention of participant having a negative experience while being screened for cervical cancer	E: Nunca dijeron, osea que decían de que porque se le subía la presión que porque se le bajaba la presión y por esto y por aquello, siempre por excusa y siempre nos ponemos a pensar por eso es que le terminó avanzando la enfermedad a mi mamá, porque la tuvieron casi tres años creo yo en la capital, que venga mañana que venga tal día, que venga entre 2 meses, así la tenían en cambio aquí cuando se fue a hacer los exámenes en Sarstun en tal día la vamos a operar y entonces así fue como se detectó que tenía cáncer. Cuando se le detectó todos nosotros nos derrumbamos para que mentirle, pero por detectarlo luego ella está bien de salud, gracias a Dios
	Communication	SE_COM	Any mention of communication or lack of communication between participant and their provider during cervical cancer screening	E: Sí, pero bien por ejemplo llegan en cualquier lugar, con un doctor pero solo les dicen un poco nada más, un poco de información, no es igual que darle charlas o dar in formación más amplia
	Support	SE_SUP	Any mention of support or lack of support between participant and their provider during cervical cancer screening	Entrevistada: Si porque a veces ha pasado eso; hasta a mí me lo han hecho. Entonces a veces la gente prefieren a gentes conocidas y a veces tratan mal Entrevistada: Porque yo llegué el día sábado. Llegue como a las ocho o nueve de la mañana. Pero… le dije a la enfermera ¨-Es que quiero entrar a la emergencia-¨. -¨Y qué tienes- me dijo.¨ ¨-Es que ya no aguanto- le dijo yo, y ya mañana es domingo y está el lunes sería. Por favor le dije, es una emergencia¨. ¨-Ah no, si ya tenes un día de esperar aguantá otro más-¨
	Cultural competence	SE_CC	Any mention of cultural competence or lack of cultural competence between participant and their provider during cervical cancer screening	E: Se le queda un poco el espéculo cuando llega a la matriz porque yo, ellos estaban explicando con la otra, hay una enfermera para explicarle ahí. Y la enfermera que me hizo el Papanicolaou le explicó a ella que no puede entrar, y ya hacía rato que estoy acostada ya me duele la vagina, como yo lo he hecho solo un rato y no duele mucho, pero ese día me dolió mucho, no entrae el espéculo, no alcanza el útero talvez así estaban diciendo ellos, ellos pensaronba que no entiendo más el español, entonces estaban explicando la enfermera que trabaja ahí le estaba explicando a la enfermera practicante, no se puede entrar el espéculo, que vamos a hacer, no sé, no sé… mejor lo dejamos así, y ella está bien en su.. no sé qué miraron ahí, está bien
	Privacy	SE_PRI	Any mention of the autotoma as a private experience	
	Confidentiality	SE_CONF	Any mention of the autotoma as a confidential experience	
	Ease of Use	SE_EASE	Any mention of the ease of use of the autotoma	
	Comfort	SE_COMF	Any mention of the autotoma as a comfortable experience	
	Concerns	SE_CON	Any mention of participant’s concerns about completing the autotoma	E: Bien yo pienso que si la pueden tomar, al menos yo, yo si a mí me explicarán cómo hacerlo correctamente los pasos que hay que hacerlos si, porque yo al menos yo digo, será que lo estoy haciendo bien, entonces ese es mi miedo
*Cervical cancer knowledge*				
	Previous knowledge	CC_PK	Any mention of participant having any information knowledge of cervical cancer	…primero que le da una infección y se convierte ya poco a poco va avanzando… … la enfermedad va por grados…
	No knowledge	CC_NK	Any mention of participant having no information or knowledge of cervical cancer	K: Pero antes de la investigación, antes de que vinieron a su casa, había escuchado de…? E: No K: ¿Nunca? E: No
	Misinformation	CC_MI	Any mention of participant having incorrect information or knowledge of cervical cancer	K: ¿Ha escuchado de unas pruebas de detección para este cáncer… del cuello de matriz? E: No K: ¿Ha escuchado del Papanicolaou? E: Sí, del Papanicolaou si
*HPV knowledge*				
	Previous knowledge	HPV_PK	Any mention of participant having any information knowledge of HPV	“Eso dicen se contagia cuando uno tiene relación con hombres, dicen… Sólo eso se de ese Papiloma”
	No knowledge	HPV_NK	Any mention of participant having no information or knowledge of HPV	K: Ah okey. Sí, tiene sentido de que no sale la conversación, uno no va hablar sobre estos temas. Eh… Bueno hemos hablado un poco sobre el cáncer del cuello de matriz. ¿Ha escuchado usted sobre el virus del VPH o de papiloma humano? E: No
	Misinformation	HPV_MI	Any mention of participant having incorrect information or knowledge of HPV	K: ¿Y no dijeron algo más sobre este virus, sólo que se contagia de persona a persona? E: Sí, a veces se contagia en el baño dicen el doctor, ¿cómo se llama eso.? (Habla en Tzutujil) T: por los baños públicos, por las tazas
*Screening knowledge*				
	Previous knowledge	SK_PK	Any mention of participant having information or knowledge of cervical cancer screening	E: Había escuchado del cáncer del cuello de la matriz que es si uno no está haciéndose periódicamente su Papanicolaou… no se puede detectar porque es algo silencioso… qué no da ningún síntoma y ya esta ahí, entonces cuando uno se da cuenta ya ha avanzado bastante
	No knowledge	SK_NK	Any mention of participant having no information or knowledge of cervical cancer screening	Entrevistada: Venía más abajo. Ajá. Y entonces el doctor me dijo, puede ser que tienes un problema allí en la matriz. Mejor te voy a sacar, te voy a hacer un papanicolaou si no te molesta o estas de acuerdo, me dijo. Entonces yo le dije, ¿qué es eso?, nunca me han hecho eso
	Misinformation	SK_MI	Any mention of participant having incorrect information or knowledge of cervical cancer screening	Entrevistadora. ¿Entonces, eh, la relación entre no tener como marido y el papanicolaou es que por no tener esas relaciones le duele más? Entrevistada: Ajá. Sí Entrevistadora: Ahh… ok Entrevistada: Según los comentarios pero yo no pienso así. Es igual digo yo. Teniendo pero ya mi cuerpo ya cambió porque yo ya soy una mujer que es diferente que ser una señorita. Ellas, así es diferente
*Knowledge source*				
	Provider	KS_PRO	Any mention of participant obtaining knowledge about cervical cancer or HPV from her provider or health care center	E: Eso fue, en una hoja que en un tiempo habían repartido y uno empezó a leer las hojas que decían… K: ¿Y dónde obtuvo las hojas? E: Era un tiempo en que vinieron unos doctores de allá y empezaron a regalar la hoja
	Radio	KS_RAD	Any mention of participant obtaining knowledge about cervical cancer or HPV from the radio	“Interprete: Si, ella ha escuchado del cáncer de la matriz por medio de la radio y que ella se preocupa por ella misma porque ella dice que durante el tiempo que está casada con su esposo no la ha tomado en cuenta no la ha vitaminado entonces ella tiene miedo de tener cáncer en la matriz, y ella dice que cuando nació su hija, la que está aquí, tenía un día de nacido cuando ella empezó a trabajar otra vez con la mostacilla porque no tien el apoyo de su esposo.”
	Television	KS_TEL	Any mention of participant obtaining knowledge about cervical cancer or HPV from the television	E: Ah por los malestares que yo sentía, yo más o menos sospechaba que podría ser, pero no porque yo había vivido esa experiencia en mi casa o con algún vecino cerca sino por lo mismo que a mi siempre me ah gustado escuchar programas, cuando miro reportajes de salud me gusta pasar mis ojos para leerlo, y aun por medio de la televisión, cierto día yo ví no sé si era película, era drama no sé sobre como la persona se estaba sintiendo, entonces cuando yo empecé a sentir esos malestares, me puse a pensar que podría ser… verdad? Pero solamente así, porque yo solo me recordaba de las escenas que yo había visto… y la dramatización que se estaba haciendo… pero cuando me vieron me dieron la noticia… me dijeron bueno si ya era sí, me dijeron que había personas que resistían el tratamiento otras que no… entonces de igual manera yo simplemente confié en Dios de que el es el que… dejarme y que los médicos hicieran lo que quisieran hacer y yo de reaccionar positivamente a lo que se me iba a aplicar
	Friend	KS_FR	Any mention of participant obtaining knowledge about cervical cancer or HPV from friends	Entrevistada: No. Solo que dijo que, que ella tenía cáncer pero yo no me explico, eh, por, por, por qué tendría ese cáncer. O, según ella dice que solo tuvo ese cáncer como dos años. Pero yo sin preguntarle como es algo pues que yo no tengo derecho de preguntarle, pero ella me dijo que por medio de que ese cáncer, de que ese cáncer entra es porque uno no se cuida. O porque puede tener eh relaciones uno con varios hombres según ella. Pero le yo, yo no puede decir o yo no entiendo por qué, porqué entra esa enfermedad. Y… ¿le puedo hacer una pregunta?
	Family	KS_FA	Any mention of participant obtaining knowledge about cervical cancer or HPV from family	I: ¿Y donde obtuvo usted esta información? E: Pues, como mi abuela paterna padeció de esto
	Jornada	KS_JOR	Any mention of participant obtaining knowledge about cervical cancer or HPV from a jornada	
	Workshops	KS_WORK	Any mention of participant obtaining knowledge about cervical cancer or HPV from workshops	E: Cómo le digo a mí me gusta meterme mucho al internet y a parte en charlas lo he escuchado también K: ¿En charlas, en las públicas o las del centro de salud? E: Las publicas K: ¿Tenían una charla sobre el VPH? ¿Qué más dijeron? No se si usted recuerde
	Internet	KS_INTER	Any mention of participant obtaining knowledge about cervical cancer or HPV from the internet	K: Entonces esto es como sobre su salud personal digamos, eh…pero sobre como información más general o sea si usted quiere usted ha escuchado de una enfermedad y quiere aprender más ¿como lo haría, de aprender más? E: Me gusta mucho meterme al internet
	Cancer experience	KS_CE	Any mention of participant obtaining knowledge about cervical cancer or HPV from person experience or experience from a friend or family member	Entrevistadora: Ah. Ok. ¿Me puede explicar un poco de lo que a escuchado usted? Entrevistada: Em… porque hace poco murió una señora, que después la familia lamentó mucho, porque la señora por vergüenza no hizo el papanicolaou, como le habían dicho los doctores. Porque es muy importante saber si uno está bien aunque uno lo ve así físicamente, pero por dentro no sabemos si estamos bien… Sí comemos pero, lamentablemente no podemos no saber si estamos perfectamente bien y la señora murió
	Print media	KS_PM	Any mention of participant obtaining knowledge about cervical cancer or HPV via print media	
	Community Health Worker	KS_CWH	Any mention of participant obtaining knowledge due to her work as a community health worker	
	Preferred method	KB_PREF	Any mention of the participant’s preferred method for receiving information about cervical cancer for HPV	
*Screening decision making*				
	Self	SDM_SE	Any mention of participant making her own decisions about getting screened for cervical cancer	T: Ella decidió ir a hacerse el examen de Papanicolaou porque tiene irregularidades en su menstruación
	Partner	SDM_PAR	Any mention of participant’s partner making her decisions about her getting screened for cervical cancer	E: Porque las mujeres de aquí, yo digo, tienen una mala costumbre cuando hacen algo tienen que pedir permiso a sus maridos, pero hay unos hombres que no les dan permiso. Entonces por eso
	Shared	SDM_SH	Any mention of participant and her partner sharing decision making about getting screened for cervical cancer	E: siempre hemos tenido conversaciones así…. Porque o sea más que todo confío en el también para conversarle las cosas… y entonces sobre lo de eso sobre la salud me siento con él y se lo platico y se lo confío entonces él es una persona que me dice, si está bueno hay que realizárselo
	Family	SDM_FA	Any mention of participant’s family other than her partner making her decisions about her getting screened for cervical cancer	
*Screening motivation*				
	Symptoms	SM_S	Any mention of a participant seeking cervical cancer screening due to symptoms	…del dolor que yo tenia, del dolor del vientre que yo tenia, entonces pore so. O puede ser también tengo algún virus o algo asi, no se uno no sabe
	Family	SM_F	Any mention of a participant seeking cervical cancer screening due to motivation from family, other than her partner	E: Pues hasta ahorita solo ella; porque ya viendo eso… No! Miento.. Eso fue como 5 años más o menos… Pues ya viendo eso ya como que cada quien se preocupa más por esas cosas. Entonces ya estamos en control más K: Ah Okey. Entonces qué hacen? Como dice que ya están como más preocupados por la salud ¿Qué mas hacen ahora, ya que…? E: Pues sí, ir al ginecólogo, verdad? Y que realizar el Papanicolaou, según el médico lo diga, bueno cada 6 meses, cada año, dependiendo de lo que el médico diga…
	Partner	SM_PART	Any mention of a participant seeking cervical cancer screening due to motivation from her partner	K: ¿Y hay una persona en su vida con quien prefiere hablar sobre esos temas de la salud? E: Con el papá de mis hijos K: ah si, ¿él es alguien que usted puede hablar sobre su salud? ¿ Y cómo son esas conversaciones? E: siempre hemos tenido conversaciones así…. Porque o sea más que todo confío en el también para conversarle las cosas… y entonces sobre lo de eso sobre la salud me siento con él y se lo platico y se lo confío entonces él es una persona que me dice, si está bueno hay que realizárselo
	Friends	SM_FR	Any mention of a participant seeking cervical cancer screening due to motivation from her friends	K: Qué bonito! Usted dijo que había escuchado que es bueno realizarse su Papanicolaou. ¿De… osea dónde escucho esté? E: No, con mis amistades, ellas si siempre iban para hacer, y yo les decía yo no voy a ir porque me da miedo
	Self-care/knowledge	SM_SC	Any mention of participant seeking cervical cancer screening due to her desire to take care of her own health	…Eso es lo que se quiere conocer el resultado del examen, si los doctors te dicen que no tienes ninguna enfermedad en la matriz uno se alegra, pero si el doctor te dice que tienes que tomar alguna medicamento hay que hacerlo para que te sanes…
	Jornada	SM_J	Any mention of participant seeking cervical cancer screening due to presence of a jornada	E: No porque aquí en Livingston, de repente ponen los cartelones ahí en el centro de salud. Tal día hay Papanicolaou, y nos vamos, ay veces se lo hacen hasta 50 a 40 personas
	Provider	SM_P	Any mention of a participant seeking cervical cancer screening due to motivation from her provider	E: Sí porque cuando tuve mi primer hijo que fue a los 27 años, me dijeron los doctores que eso es importante… para hacerlo si es posible cada año… K: ¿Quién le dijo eso? E: Yo tenía un mi doctor en Puerto Barrios
	External incentives	SM_EI	Any mention of a participant seeking cervical cancer screening due to external motivations other than those mentioned above	T: Que ella estaba haciendo beneficiada por el gobierno pasado que se les daba dinero a los niños se llama Bono Seguro pero que a ella las obligaron a hacer el Papanicolaou
	Cost	SM_COST		
*Screening barriers*				
	Embarrassment	SB_EMB	Any mention of personal embarrassment as a barrier to screening	Antes aquí en Santiago, Atitlan las mujeres casi no iban con los doctores, por vergüenza que les vienen…
	Stigma	SB_STIG	Any mention of judgement from others as a reason barrier to screening	“pero hay personas que no han entendido, no quieren por vergüenza, por vergüenza no cuentan de ellas, en cambio yo no tengo vergüenza de contar de mi porque quiero que me ayuden
	Confidentiality	SB_CON	Any mention of personal lack of confidentiality as a barrier to screening	E: Pues porque pienso que a veces acá en el centro de salud como que se va perdiendo un poco lo que es la ética, entonces ya como nos conocemos… ya como que no, ya no hay mucha confianza en ese sentido
	Economic	SB_ECO	Any mention of lack of economic resources as a barrier to screening	…a veces también hay escasez de dinero… Se va al CAIMI y paga taxi, se paga picop y a veces la gente aquí casi no tiene la posibilidad de ir y entonces, ya con eso dicen –Mejor no que gastar e ir alla, mejor no, y si no me atienden, mejor no
	Logistical	SB_LOG	Any mention of lack of logistical barriers (i.e. time, childcare) as a barrier to screening	… Por el trabajo, con los hijos, y las cosas que uno hace en la casa, como ama de casa [en referencia a que no pueden ir al centro de salud]
	Provider Treatment	SB_PROT	Any mention of poor treatment by a provider as a barrier to screening	…a veces tratan mal [centro de salud]
	Provider Gender	SB_PROG	Any mention of gender of provider as a barrier to screening	K: ¿Y usted tiene una preferencia de donde hacer el Papanicolaou? E: No K: Sólo que si usted está … E: Sólo que prefiero que sea una mujer que me lo haga K: ¿Una mujer? E: Aja
	Partner	SB_PAR	Any mention of a participant’s partner stopping or not allowing her in some way to be screened	T: Ella dice que hay hombres que a veces se entienden que es necesario para las mujeres hacerse este examen y que hacen todo lo posible para que sus esposas estén bien pero en el caso de ellas, su esposo no es así
	Family	SB_FA	Any mention of a participant’s family stopping or not allowing her in some way to be screened	
	Misinformation	SB_MI	Any mention of a participant’s lack of knowledge or information as a barrier to screening	T: Que ellas las aconseja que a que se realicen este Papanicolaou porque solo dura un rato, porque ella cuando se hizo el examen del Papanicolaou hubo unas mujeres que le preguntaron, que si es cierto que se tiene que sacar toda la matriz, y se ponen en una mesa como dicen toda las personas, y ella dijo que no es cierto, que solo es un poquito, por que no es posible que se saque todo
	Symptoms	SB_PR	Any mention of a participant’s lack of symptoms as a reason for not being screened	Entrevistada: No, no hay ninguna razón. Simplemente porque uno dice que se siente bien y está bien y uno dice que uno está bien físicamente. Y para qué va ir hacer un papanicolau y si uno lo tiene que pagar y quita tiempo. Tal vez nosotros pensamos entre en persona porque nosotros trabajamos en casa y no podemos salir por los hijos. Y así…
	Fear of results	SB_FEAR	Any mention of a participant’s fear of her results as a barrier to screening	
	Language/culture	SB_LAN	Any mention of a participant’s language or culture of her results as a barrier to screening	“Si, es por eso porque antes a mi no me pusieron a estudiar si pudiera yo misma le contaría sobre mí pero tienen que traducir lo que digo, eso lo que ha costado más no sé si para ella es lo mismo como lo veo yo¨ Aquí hay enfermeros que hacen la traducción pero lo que veo es que es vergonzoso porque es tu paisano quien hace el Papnicolaou¨
	Fear of procedure	SB_DIS	Any mention of a participant’s fear of the procedure as a barrier to screening	E: Pues cuando fui, con un poco de miedo, verdad, porque había dicho que duele hacer el Papanicolaou, pero fui, con miedo, si… El doctor me dijo que no duele sólo uno tiene que relajar antes de meter el pato a la mujer para mirar que lo que uno tiene
*Support system*				
	Family	SS_FAM	Any mention of the participant’s family, other than her partner, providing support to her in relation to her healthcare	K: Aja, y ¿con quien… si usted podría escoger a una persona para hablar sobre su salud, digamos personal como de mujer… a quién escogería? E: A mi mamá K: Su mama? Me podría explicar un poco ¿Por qué escogería a su mamá? E: Porque, tal vez, ella es mujer y ha tenido otras experiencias, talvez ella me puede decir mirá…o me puede recomendar algo por su experiencia que ella tenga
	Friend	SS_FRI	Any mention of the participant’s friend(s) providing support to her in relation to her healthcare	K: Me alegro! Usted habla con alguien sobre estos temas de su salud, de mujer, digamos? E: No K: Nunca, en el pasado ha hablado con alguien? Si podría escoger a una persona para hablar sobre estos temas ¿a quien escogería? E: A una amiga K: A una amiga, y ¿porqué una amiga? E: Hay amigas que si son de confianza…
	Partner	SS_PAR	Any mention of the participant’s partner providing support to her in relation to her healthcare	K: ¿Y hay una persona en su vida con quien prefiere hablar sobre esos temas de la salud? E: Con el papá de mis hijos K: ah si, ¿él es alguien que usted puede hablar sobre su salud? ¿ Y cómo son esas conversaciones? E: siempre hemos tenido conversaciones así…. Porque o sea más que todo confío en el también para conversarle las cosas… y entonces sobre lo de eso sobre la salud me siento con él y se lo platico y se lo confío entonces él es una persona que me dice, si está bueno hay que realizárselo
	Community	SS_COM	Any mention of the participant’s community providing support to her in relation to her healthcare	E: Él nos apoyó, el alcalde que acaban de quitar ahorita, que se llama Chente. Él era la persona que nos terminaba apoyando sobre las quimioterapias de mi mamá K: Y es como un amigo, o es algo normal E: Es alcalde de aquí, es algo normal de apoyar a las personas del pueblo K: Ah okey, es como un programa, o es algo informal que hace E: Es algo formal por ser alcalde K: okey entonces parte de su trabajo es apoyar E: Aja apoyar a las personas del pueblo K: ¿Y como apoyó en que sentido? E: En el sentido de que él nos terminó apoyando a nosotros sobre el dinero, nos terminaba dando Q2,000 y nosotros podíamos aportar lo demás. Que no fue por mucho tiempo pero si nos ayudo en algo
*Traditional medicine*		TM	Any mention of a participant using traditional medicine in any context	Entrevistada: Pues ella me dice aha… como mujer a mujer nos comunicamos y hablamos decimos que tal vez por el frío que te da esto tenes dolor, tómese… como digamos tomillo cocido con hojitas de… como se llama esto… naranja o… o limón. Entonces, con eso, uno lo cose y despues lo toma entonces de ahi le pasa el dolor a uno. O tal vez este manzanilla, ajenjo, de eso casi nos tomamos porque somos mujeres para poder tener la matriz… nosotros hablamos así para tenerlo asi no fría. Aha…. tal vez por el frío nosotros… se enfría nuestro estómago. Tal vez por eso. Y solo asi nos tomamos algo y nos sentimos un poco relajados y así nos acostamos un poco, y si tenemos acetaminofén con uno nada más, o ibuprofeno. Y asi por que… como le digo, para ir a un doctor es mucho
*Cancer experience*		CE	Any mention of the participant or a loved one having experienced cancer	
*Auto-toma experience*		ATE		
	Positive experience	ATE_PE	Any mention of the participant having a positive experience using the autotoma	E: Sí me hice la autotoma K: Y cómo fue esta experiencia con la autotoma E: Bien, me gustó, no fue incómodo fue cómodo, rápido, fácil y sencillo
	Negative experience	ATE_NE	Any mention of the participant having a negative experience using the autotoma	
	Privacy	ATE_PRI	Any mention of the autotoma as a private experience	K: En casa! ¿Y porqué le gusto de que lo hizo en casa? E: Es por que uno mismo, que uno mismo… así sola que hizo sola…
	Confidentiality	ATE_CONF	Any mention of the autotoma as a confidential experience	E: De que esto es algo nuevo para acá para Livingston. Es algo nuevo y es más confidencial porque uno no va con un médico, uno mismo se lo hace en casa
	Ease of use	ATE_EASE	Any mention of the ease of use of the autotoma	E: y traté de seguir las instrucciones para hacerlo correctamente porque a veces también… como era mi primera experiencia, podría equivocarme a la hora de usar el aparato pero era muy sencillo de usar, entonces me gusto bastante la experiencia
	Comfort	ATE_COMF	Any mention of the autotoma as a comfortable experience	E: pero lo desagradable fue donde si sentí un poco que me lastimó con los cepillos, seguramente estaba raspandome, entonces si fue algo incómodo eso, pero sí de ahí luego de eso no tuve dolor o molestias o sangrados o cosas así…no? Sólo fue esa molestia de raspar, creo que en cualquier parte de mi piel también lo hubiera sentido, porque cómo es sensible, la vagina y el útero y sentir eso, el cepillo era áspero y sentir que te está raspando
	Hesitancy	ATE_HES	Any mention of the participant being hesitant to complete the autotoma	Entrevistada: Un poco pero…También me dijo la muchacha que vino, me dijo: ¨-Solo hay que respirar profundo y relajarse y no tener miedo porque a veces uno lo hace que duela-, me dijo¨. Y sí casi no me dolió; un poquito pero después eh estaba con nervios entonces después me relajé mejor y sí todo salió bien
	Refusal	ATE_REF	Any mention of the participant refusing to complete the autotoma	E: Osea… que me habían dado… para hacerme el examen pero ese día yo no acepté, pero para la próxima después del ultrasonido que voy a ir a hacerme talvez me arriesgo porque ese día yo no quise… K: ¿Y me puede decir un poco de porque no quiso hacer el autotoma? E: Porque no estaba… no lo había pensado muy bien todavía
	Concerns	ATE_CON	Any mention of participant’s concerns about completing the autotoma	E: Bien yo pienso que si la pueden tomar, al menos yo, yo si a mí me explicarán cómo hacerlo correctamente los pasos que hay que hacerlos si, porque yo al menos yo digo, será que lo estoy haciendo bien, entonces ese es mi miedo
	Control	ATE_CONT	Any mention mention of the participant being in control while completing the autotoma	Entrevistada: Sí, bueno, yo me sentía que yo era la doctora. Ajá. Porque sin necesidad de un doctor, bueno, yo me sentía así pues. A lo mejor, va, un ejemplo; digamos que yo soy la doctora que ahorita me checo a mí misma. Ajá. Y me saqué esa prueba. Va, yo sentí eso fue pues
	Speed	ATE_SPEED	Any mention of speed with which the participant could or did complete the autotoma	
*Community health workers*		CHW		
	Trust	CHW_TRU	Any mention of the participant expressing their trust in the community health workers	E: como uno confía en las personas que son de aquí, verdad… y yo le dije que sí me lo iba a hacer por cualquier cosa
	Personal familiarity	CHW_PF	Any mention of the participant having personal familiarity with any of the community health workers	E: Bueno, como ellas me explicaron que era, hasta incluso la muchacha la morenita que me lo vino a hacer… yo la conozco, entonces me dijo que ella ya se lo había hecho de que no era nada malo y
*Motivation*		MO		
	Self-care	MO_SC	Any mention of self-care as motivation for completing the autotoma	T: Ella se puso feliz cuando le hicieron este examen porque quería saber como estaba de la matriz porque hay veces que a ella le duele el estómago y le preocupase pregunta que por estar sentada todo el tiempo por haciendoer mostacilla le afecte la matriz o tenga algo en la matriz
	Perceived risk	MO_PR	Any mention of perceived risk of cervical cancer as motivation for completing the autotoma	E: Un poco talvez… un 20%, pero no tanto por mí sino que el papá de mis hijas es un hombre…era un hombre bastante infiel, entonces por esa misma razón yo decidí terminar la relación con él, porque tuve miedo de que pudiera el contraer alguna infección de transmisión sexual y afectarme a mí
	Cost	MO_COST	Any mention of low-cost as motivation for completing the autotoma	Entrevistada: Yo le dije, porque ella estaba allá en la casa y le dije: ¨-A que bueno que, que, que lo hiciste-, le dije, -porque estando en casa. ¡Que bueno!-, le dije. -¿Y cuánto cobran?-¨. ¨- Pues nada-, me dijo¨. ¨—Que bueno-, le dije yo¨. (Risas)
	Convenience	MO_CON	Any mention of convienence as motivation for completing the autotoma	E: No sé, hay otras que si no les encanta, pero yo voy en el sentido de que ya vinieron hasta acá para uno poder hacerse el examen, entonces uno ya no puede ya no es necesario irse corriendo para irse a otro lado, para irse a hacerse los exámenes. Entonces eso es lo que más me encantó
	Self-administered	MO_SA	Any mention of the self-administration of the test as motivation for completing the autotoma	Entrevistada: Sí yo lo ví, yo lo ví. Entonces que el aparato que tenía le entregaron a ella. Ella lo fue a hacer en el baño. Entonces por eso dije: ¨-Que bueno que uno lo haga-¨
	Knowledge		Any mention of the desire for more information or knowledge as motivation for completing the autotoma	E: No, solo que, que bonito que estén realizando esto porque le dan más información a uno, entonces para mi está muy bien, y me gustó participar porque… está uno más abierto a la información, y talvez uno más se informa de cosas que uno no sabía, verdad, que bien que lo estén haciendo para ayudar a más gente, verdad
	Symptoms	MO_S		
*Future self-screening*		FS	Any mention of the participant obtaining any type of screening for cervical cancer in the future	Entrevistadora: ¿Sí? Perfecto. ¿Y cómo sentiría usted de, de hacer esta prueba otra vez? Entrevistada: Pues, eh, pues así como me sentí la primera vez así me volvería a sentir si me lo voy a hacer de nuevo…
